# Uncovering the Role of Direct Oral Anticoagulants in Stroke Prevention for Atrial Fibrillation: A Review of the Literature

**DOI:** 10.7759/cureus.63675

**Published:** 2024-07-02

**Authors:** Sudipta Rao, Shailesh Aggarwal, Sweatha Mani, Abirami Balasubramanian, Keerthana Veluswami

**Affiliations:** 1 Internal Medicine, JSS Medical College, Mysore, IND; 2 Internal Medicine, K.A.P. Viswanatham Government Medical College, Tiruchirappalli, IND; 3 Internal Medicine, Stanley Medical College and Hospital, Chennai, IND

**Keywords:** warfarin, vitamin k antagonists, stroke, pharmacodynamics, direct oral anticoagulants, atrial fibrillation

## Abstract

Atrial fibrillation (AF) is a predominant contributor to morbidity and mortality, and stroke prevention remains the mainstay for the management of AF. The precise mechanism involved in thrombus formation remains unknown. However, factors such as stretch-induced fibrosis, endothelial dysfunction, disordered atrial contractions, and pro-thrombotic states have been postulated for the development of AF. Various risk assessment strategies have been acknowledged for determining the risk of stroke in AF, of which the congestive heart failure, hypertension, age ≥75, diabetes, stroke, vascular disease, age between 65-74, and female sex (CHA2DS2-VASc) score remains the ultimate risk stratification tool. For the longest time, vitamin K antagonists (VKA) were the only oral anticoagulants available but were associated with an increased risk of bleeding. Recently, direct oral anticoagulants (DOACs) were approved and considered more efficient and safer than or as secure as warfarin in stroke prevention and lowering intra-cranial bleeding events. The pharmacodynamics and pharmacokinetics of DOACs were also clarified in this article. This review article compiles current evidence-based data on the role of DOACs, uncovering their underlying mechanisms, and comparing their efficacy with warfarin in stroke prevention in AF.

## Introduction and background

Atrial fibrillation (AF) is adults' most prevalent cardiac arrhythmia, defined by irregular and disorganized electrical activity that disrupts the heart's normal sinus rhythm [1]. AF has been known for 100 years, but 1905 was a momentous year. William Einthoven (1860-1927) was the first to publish an electrocardiogram showing AF in 1906 [2]. Arthur Cushny, a pharmacologist, cardiologist Thomas Lewis, and two Viennese physicians, Rothberger and Winterberg, established a relationship between pulse irregularity and AF [3]. It is believed to affect over 30 million people worldwide [4]. More than five million people in the United States (US) have AF [5]. It is anticipated that one in three White individuals and one in five African American individuals will develop AF in their lifetime [6]. Although African American and Asian individuals have higher comorbidities, the prevalence and incidence of AF are seen more in people of European ancestry [7]. Studies have shown the prevalence of AF increases with advancing age. A Scottish study revealed the incident rates per 1000 people were 0.5 for those aged 45-54 years, 1.1 for those aged 55-64 years, 3.2 for those aged 65-74 years, 6.2 for those aged 75-84 years, and 7.7 for those aged 85 years and older [8]. There is conflicting data as to whether sex plays a role in the association between risk factors and AF.

AF is a multifactorial entity. Besides non-modifiable risk factors such as age, ethnicity, and genetics, modifiable risk factors such as systemic hypertension, diabetes, alcoholism, thyroid disorders, obesity, chronic obstructive pulmonary disease (COPD), venous thromboembolism, ischemic heart disease, and valvular heart disease have been shown to bring forth structural and electrical remodeling of the atria [9]. The majority of studies have illustrated that more than 50% of patients with AF have underlying coronary artery disease (CAD), as determined by invasive or computed tomography and coronary angiography, thus revealing an undeviating association between AF and CAD [10]. The main pathogenic mechanisms predisposing an individual with CAD to develop AF are endothelial dysfunction and inflammation, thus culminating in a vicious cycle leading to an increased burden of both diseases [11]. Further to substantiate their correlation, an inflammatory biomarker named high-sensitivity C-reactive protein (hs-CRP) elevated in both AF and CAD contributed significantly to higher thrombogenesis and resultant stroke when coexisted together as opposed to AF occurring alone [12]. In spite of extensive research, uncovering the precise mechanisms for AF remains challenging [13]. Modern literature proposes that factors such as stretch-induced fibrosis, epicardial adipose tissue (EAT), chronic inflammation, autonomic nervous system (ANS) imbalances, and genetic mutations contribute incredibly to AF pathogenesis [14]. However, specific treatments targeting the root cause of AF are still unknown. Common symptoms associated with AF include palpitations, dyspnea, chest discomfort, fatigue, and dizziness, which can diminish the quality of life and increase the risk of morbidity and mortality [15]. Over a while, electrocardiograms (ECGs) have emerged as the gold standard for AF diagnosis. Recent advances in wearable devices have reinforced AF diagnosis by offering continuous, non-invasive heart rhythm monitoring, significantly contributing to health providers' detection of AF at an earlier stage [16]. The essence of AF management highlights the control of symptoms, further segmented into rate control and rhythm control, along with diminishing the risk of thromboembolism. An integrated approach to patient care is crucial for this management. Stroke prevention is achieved through anticoagulation therapy by a congestive heart failure, hypertension, age ≥75, diabetes, stroke, vascular disease, age between 65-74, and female sex (CHA2DS2-VASc) score, which particularly holds significance for patients developing stroke or systemic embolism (SE) [15]. 

Through this review, we utilize present-day knowledge and ongoing research revolving around the appropriateness of direct oral anticoagulants (DOACs) for the prevention of thromboembolism as a long-term clinical management plan for patients with AF. This review draws attention to the benefits of implementing DOACs over vitamin K antagonists such as warfarin and explains the underlying mechanisms that aid their efficacy.

## Review

Managing AF remains a significant challenge, mainly due to the risk of cardioembolic stroke. Research suggests that the principal cause of thrombus formation in the left atrial appendage (LAA) is disordered electrical signals, uncoordinated atrial contractions, endothelial disjunction, and other thrombotic states. Thrombi formed in the LAA can detach and embolize, often aiming at the cerebral circulation, and hence strokes arising from AF-related emboli have been shown to have severe consequences compared to strokes unrelated to AF [17,18]. Further, it is important to stress that strokes associated with AF demonstrated overall severe neurological outcomes, greater cognitive dysfunction, and dementia compared to strokes without AF [19]. Various risk criteria have been postulated to assess the probability of developing stroke, or SE. In 2010, Lip and colleagues presented the CHA2DS2-VASc score as an advancement over the previously used CHADS2 score [20]. The score was simple to calculate and memorize; the primary goal was explicitly identifying individuals at high risk for thromboembolism. A peculiar feature of the score is its ability to categorize age further, appointing two points for those aged 75 and older and one for those aged between 65-74. Auxiliary risk factors include vascular disease encompassing prior myocardial infarction, aortic plaque, and arterial vascular disease. The CHA2DS2-VASc score has a slightly superior predictive value to its predecessor scores [21-23]. Currently, it is approved as a standard criterion for deciding stroke risk [24,25]. Ongoing discussions are underway about including female sex as a risk factor.

Evolution of direct oral anticoagulants

Earlier in 2009, various studies examined the capacity of oral anticoagulants with vitamin K antagonists (VKAs), such as warfarin, against placebo or aspirin for preventing AF-related stroke [26]. Every study formulated a different design, targeting specific international normalized ratio (INR) ranges; overall, a target INR range of 2.0-3.0 was urged among high-risk patients [26]. The conclusion of these studies illustrated a substantial benefit of using VKA therapy as compared to aspirin or placebo, after which either VKA therapy or aspirin was recommended for intermediate stroke risk. However, bleeding complications, including intracranial hemorrhage, were among the adverse events associated with this therapy. To counter these adverse effects, in 2010, the US Food and Drug Administration (FDA) approved its first DOAC, dabigatran, followed by rivaroxaban, apixaban, edoxaban, and betrixaban, which were compared to VKA therapy for stroke prevention in non-valvular AF. Based on their positive effectiveness and safety profile, countries such as North America and Europe sanctioned these drugs. However, specific concerns revolving around the reliability of some data from the Apixaban for Reduction in Stroke and Other Thromboembolic Events in Atrial Fibrillation (ARISTOTLE) trial comparing apixaban vs. VKA were put forward [27]. In defiance of these concerns, the US FDA, after an intensive review, approved the medication with a package label emphasizing the overall stroke and SE reduction compared to VKA therapy. These drugs exhibited superior or non-inferior standards compared to VKAs, i.e., warfarin and low molecular weight heparin (LMWHs), lowering thromboembolic rates with equivalent or decreased bleeding risk [28-31]. Later, various evidence-based studies claimed the overall potency and safety of DOACS over VKA [32,33]. Several advantages DOACs offer over warfarin include less frequent follow-ups, no prothrombin time/international normalized ratio (PT/INR) monitoring, reduced monitoring needs, quicker onset and offset (significant for pre-procedural and acute bleeding management), and decreased food and drug interactions [34]. Subsequently, this led to increased DOAC prescriptions compared to warfarin by 2013, with apixaban being the most common DOAC prescribed for patients with non-valvular AF [35]. DOACs have become the most conventional anticoagulant used in patients with AF; specific concerns regarding their strict compliance and pertinacity to therapy still exist [36]. Analysis from Ontario, Canada, revealed that one-third of the patients prescribed either rivaroxaban or dabigatran were no longer taking it following six months of initiation [37]. Elevated rates of stroke and mortality were observed among these patients compared to those who continued taking DOACs as prescribed.

**Figure 1:**
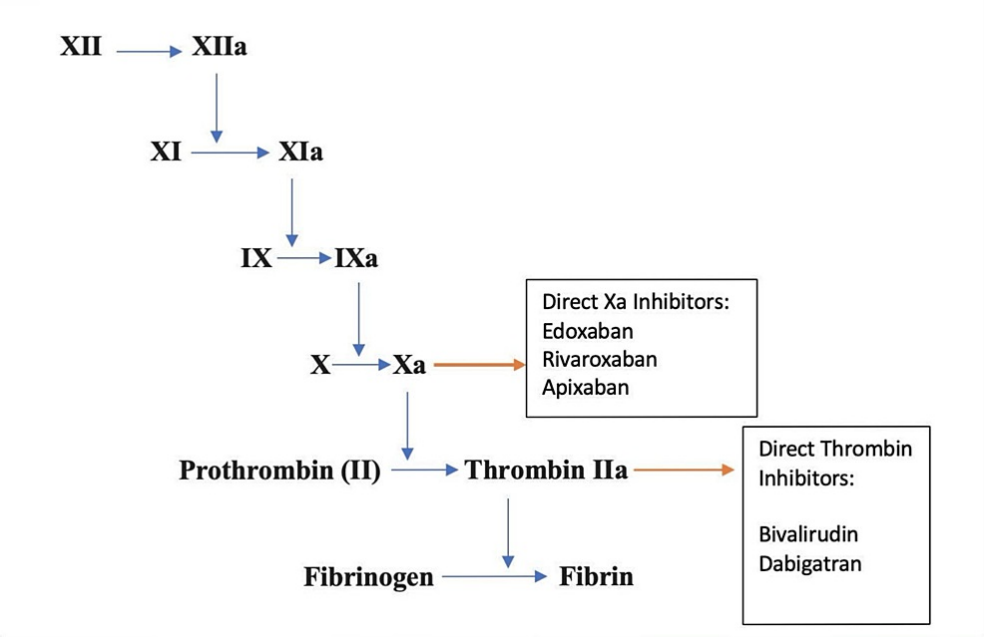
Mechanism of action of DOACs DOAC: direct oral anticoagulants Image Credits: Sudipta Rao

DOACs are categorized into two main groups. Figure 1 gives an insight into their mechanism of action.

Apixaban, a direct oral factor Xa inhibitor, has prompt absorption, a half-life of 12 hours, and 25% excretion through the kidney [38]. Apixaban achieves its maximum concentration (Cmax) three to four hours after oral administration [39]. Rivaroxaban (Xarelto), a competitive inhibitor of factor Xa, was first approved by the FDA after its study in the Rivaroxaban Once Daily Oral Direct Factor Xa Inhibition Compared with Vitamin K Antagonism for Prevention of Stroke and Embolism Trial in Atrial Fibrillation (ROCKET AF) trial [40]. Apixaban and rivaroxaban use with either cytochrome P450 family 3 subfamily A member 4 (CYP3A4) inducers or inhibitors should be avoided [41]. Their drug is edoxaban, categorized as a reversible Xa inhibitor [42]. Edoxaban endures transport through P-glycoprotein (P-gp). Hence, the simultaneous use of potent glycoprotein inhibitors such as verapamil and quinidine can inhibit edoxaban metabolism and increase its toxicity [43]. Dabigatran, a direct thrombin inhibitor, was the first DOAC approved by the FDA based on the reports of the RE-LY (Randomized Evaluation of Long-term Anticoagulant Therapy with Dabigatran Etexilate) trial [44]. It has a bioavailability of 6-7%, which is relatively low [45]. Based on these pharmacological properties, taking these medications with meals is recommended to allow for greater absorption and higher bioavailability. The following table briefly describes the pharmacokinetics and pharmacodynamics of DOACs (Table 1).

**Table 1:** Pharmacokinetics and pharmacodynamics of DOACs P-gp: P-glycoprotein; CYP3A4:cytochrome P450 family 3 subfamily A member 4; Mg:milligram; DOACs: direct oral anticoagulants

Characteristics					
Drug Name	Bioavailability	Half Life	Renal Excretion	Hepatic Excretion	Drug Interactions
Apixaban	<50% for 10 mg dose	<12 hours	<25%	Yes	Inhibitors of CYP3A4 and P-gp
Rivaroxaban	66%	6-12 hours	35%	Yes	Inhibitors of CYP3A4 and P-gp
Edoxaban	<60% for 60 mg dose	6-11 hours	<50%	No	Inhibitors of P-gp
Dabigatran	6-7%	11-14 hours	85%	No	Inhibitors of CYP3A4 and P-gp

Summary of DOAC trials

The summary of the following meta-analyses, RE-LY, ROCKET AF, ARISTOTLE, Effective Anticoagulation with Factor Xa Next Generation in Atrial Fibrillation-Thrombolysis in Myocardial Infarction Study 48 (ENGAGE AF-TIMI), and EXPLORE Xa, was compared with dose-adjusted warfarin (INR 2-3) in Table 2 and Table 3.

**Table 2:** Summary of DOAC trials ECG: electrocardiogram; SE: systemic embolism; ENGAGE-TIMI 48: Effective Anticoagulation with Factor Xa Next Generation in Atrial Fibrillation-Thrombolysis in Myocardial Infarction Study 48; ARISTOTLE: Apixaban for Reduction in Stroke and Other Thromboembolic Events in Atrial Fibrillation; RE-LY: Randomized Evaluation of Long-Term Anticoagulation Therapy; ROCKET AF: Rivaroxaban Once Daily Oral Direct Factor Xa Inhibition Compared with Vitamin K Antagonism for Prevention of Stroke and Embolism Trial in Atrial Fibrillation: BID: two times a day; DOAC: direct oral anticoagulant

Reference	Study	Study Design	Comparison	Drug and Dosage Used	No. of Cases	Primary outcome	Duration of Follow-up	Diagnostic Criteria	Conclusion
Robert P. Giugliano et al. (2013) [43]	ENGAGE AF TIMI 48	Randomized, double-blind, double-dummy trial	Edoxaban. Vs warfarin	Edoxaban low dose: 30mg BID, Edoxaban high dose: 60mg BID	21,105	Stroke and SE	2.8 years	Twenty-one years or older, ECG findings and CHADS2 score of 2 or more.	Edoxaban was non-inferior compared to warfarin in the reduction of stroke and SE.
Stuart J Connolly et al. (2013) [46]	EXPLORE -Xa	Randomized control trial	Betrixaban vs warfarin	Betrixaban 40,60,80 mg OD	508	Major or clinically relevant non-major bleeding	147 days	18 years of age or older; ECG findings; one or more risk factors for stroke.	Betrixaban was associated with equivalent to or lower risk of bleeding as compared to warfarin.
Christopher B et al. (2011) [41]	ARISTOTLE	Randomized, double-blind trial	Apixaban vs warfarin	Apixaban 5mg BID	18,201	Ischemic or hemorrhagic stroke and SE	More than 12 months	21 years of age or older ECG findings one risk factor for stroke CHADS2 score of 2 or more.	Apixaban outperformed warfarin in the prevention of SE and stroke.
Manesh R. Patel et al. (2011) [40]	ROCKET AF	Randomized, double-blind, double-dummy, event-driven trial	Rivaroxaban vs warfarin	Rivaroxaban 20 mg OD	14,264	Stroke and SE	More than 14 months	Moderate/ high risk for stroke ECG findings CHADS2 2:10% CHADS2 ≥3: 90%.	Rivaroxaban was non-inferior to warfarin in prevention of stroke and SE.
Stuart J et al. (2009) [44]	RE-LY	Randomized control trial	Dabigatran vs warfarin	Dabigatran 110mg BID High dose:150mg BID	18,113	Stroke and SE	2 years	ECG findings, one risk factor for stroke CHADS2 0–1: c. 32% CHADS2 2: c. 35% CHADS2 ≥3: c. 33%.	Low-dose dabigatran revealed similar rates of stroke and SE. Lower rates of stroke and SE were associated with high dose dabigatran.

**Table 3:** Effects of different DOACs NA-Not Applicable; DOAC: direct oral anticoagulants; RE-LY: Randomized Evaluation of Long-Term Anticoagulation Therapy; ROCKET AF: Rivaroxaban Once Daily Oral Direct Factor Xa Inhibition Compared with Vitamin K Antagonism for Prevention of Stroke and Embolism Trial in Atrial Fibrillation; ARISTOTLE: Apixaban for Reduction in Stroke and Other Thromboembolic Events in Atrial Fibrillation; ENGAGE-TIMI 48: Effective Anticoagulation with Factor Xa Next Generation in Atrial Fibrillation-Thrombolysis in Myocardial Infarction Study 48

Drug Name	Study Name	Relation with Stroke/Systemic Embolism	Mortality	Adverse Effects		
				Major Bleeding	Gastrointestinal Bleeding	Intracranial Bleeding
Dabigatran 110 mg BID	RE-LY [44]	Non-Inferior	Equivalent	Decreased	Equivalent	Decreased
Dabigatran 150 mg BID		Superior	Equivalent	Increased	Increased	Decreased
Rivaroxaban 20 mg OD	ROCKET AF [40]	Non Inferior	Equivalent	Equivalent	Increased	Decreased
Apixaban 5 mg	ARISTOTLE [41]	Superior	Decreased	Decreased	Equivalent	Decreased
Edoxaban 30 mg OD	ENGAGE AF TIMI-48 [43]	Non Inferior	Equivalent	Decreased	Decreased	Decreased
Edoxaban 60 mg OD		Non Inferior	Equivalent	Decreased	Increased	Decreased
Betrixaban 40 mg OD	EXPLORE Xa [46]	Lower	None	None	NA	NA
Betrixaban 60 mg OD		Equivalent	None	None	NA	NA
Betrixaban 80 mg OD		Equivalent	Not significant	Not significant	NA	NA

In the ARISTOTLE trial, apixaban, a direct oral factor Xa inhibitor, was compared to warfarin. Apixaban, given at a dose of 5 mg two times a day (BID), outperformed warfarin in preventing stroke and SE, causing less major bleeding, intracranial bleeding events, and lower mortality [43]. A study done by Alexandar T. Cohen et al. also demonstrated a lower risk of recurrent VTE (HR (95% confidence interval (CI) 0.72 (0.67-0.78)), major bleeding (HR (95% CI) 0.70 (0.64-0.76)), and clinically relevant non-major (CRNM) bleeding (HR (95% CI) 0.83 (0.80-0.86)) [47]. Rivaraxoban, administered at a fixed dose of 20 mg OD, was compared with dose-adjusted warfarin in patients with non-valvular AF who were at increased risk for stroke or SE [40]. The primary outcome of the ROCKET AF revealed rivaroxaban to be as good as warfarin in preventing stroke, or SE [40].

On the contrary, apixaban (5 mg BID) and high-dose dabigatran (150 mg BID) had lower stroke and SE rates [40]. Rivaroxaban revealed equivalent rates of mortality and major bleeding, although less frequent episodes of intracranial cranial bleeding occurred in patients on rivaroxaban [41]. Edoxaban with two dose-based regimens was compared to warfarin in the ENGAGE TIMI 48 trial [43]. The primary outcome indicated that both doses of edoxaban were non-inferior to warfarin in preventing SE and stroke [43]. Edoxaban consistently showed lower rates of all types of bleeding, including primary and intracranial bleeding, except for high-dose edoxaban (60 mg BID), which exhibited increased gastrointestinal bleeding events [43]. Dabigatran, a direct thrombin inhibitor, was studied extensively in the Randomized Evaluation of Long-Term Anticoagulation Therapy (RE-LY) trial. Two fixed-dose regimens of dabigatran (110 mg BID and 150 mg BID) were compared to warfarin in patients with AF and increased risk for stroke [45]. The study concluded that low-dose dabigatran showed similar rates of SE and stroke compared to warfarin. Still, high-dose dabigatran was associated with a decreased rate of SE and stroke [45]. This difference was primarily seen in incidents of ischemic stroke, whereas hemorrhagic strokes exhibited similar rates in the two dose groups [45]. Higher major bleeding and significant hemorrhage events were noted in high-dose dabigatran; this could be attributed to the absorption of dabigatran, which requires low pH [45]. Hence, dabigatran capsules are coated with a tartaric acid core, leading to increased dyspeptic symptoms and increased gastrointestinal bleeding at higher doses [45].

On the contrary, previous studies compared another thrombin inhibitor with warfarin, named ximelagatran, which displayed similar efficacy and safety compared to warfarin but was hepatotoxic [45]. In the RE-LY trial, dabigatran was not found to be hepatotoxic [45]. Lastly, the EXPLORE Xa trial studied betrixaban, a new oral anticoagulant in the pipeline, compared to warfarin, which displayed a lower rate of SE and stroke in patients taking low-dose (40 mg) betrixaban. In contrast, betrixaban administered at 60 and 80 mg revealed a similar rate of stroke and SE compared to warfarin [46]. Betrixaban was observed to be permissible in patients with AF at increased risk of stroke [46]. Out of a sample size of 127, no individual had any major bleeding events.

In contrast, on Betrixaban 40 and 60 mg, respectively, three major bleeding events were seen on betrixaban 80 mg, which was statistically not significant (0.609 (0.145-2.557)) [41]. The primary limitation of this study is its small sample size; hence, a thorough conclusion on betrixaban's superiority over warfarin cannot be determined [46]. Another retrospective study on betrixaban, named Acute Medically Ill Venous Thromboembolism Prevention with Extended Duration Betrixaban (APEX), revealed decreased ischemic stroke rates over 77 days of follow-up [48].

Overall, the RE-LY, ARISTOTLE, ROCKET AF, and ENGAGE TIMI 48 studies revealed a significant decrease in all-cause mortality (RR 0.90, 95% CI 0.85-0.95, P = 0.0003) [49]. The EXPLORE Xa trial reported two deaths due to a vascular cause, one each in the betrixaban and warfarin groups [46]. DOACs contributed a significant reduction of 19% in stroke or systemic embolism events (RR 0·81, 95% CI 0·73-0·91; p<0·0001), compared to warfarin, which had a predominant decrease in hemorrhagic stroke [28]. Subdivision analysis catering to factors such as age, sex, prior history of stroke, renal failure, diabetes, CHADS2-score, or competency of anticoagulation with VKA unveiled no substantial relation with stroke or SE [28]. A noteworthy reduction in major bleeding events was ascertained with DOAC therapy paralleled to warfarin when the time within the therapeutic range was less than 66% (RR 0.69 vs. 0.93, P = 0.022) [28]. This indicated that the reliability and efficiency of DOACs did not depend on the ideality of warfarin treatment [28]. On the contrary, a study done by Goméz-Outes et al. demonstrated that DOACs were inferior to VKA therapy in preventing stroke or SE [49]. 

Some of the challenges met with DOACs are the higher rates of extra-cranial bleeds observed in patients with increasing age on dabigatran and rivaroxaban [28]. Major bleeding events were higher in patients aged above 80 years [28]. Hence, a dose alteration of dabigatran (110 mg BID) and apixaban (2.5 mg) is essential in individuals aged 80 years and older with comorbidities and the use of concurrent drugs, which increase bleeding risk [49]. DOACs should be avoided in patients with renal impairment [50]. Patients with less than 30 mL/min of creatinine clearance are a significant contraindication for initiating DOACs [50]. Dose alteration (dabigatran 110 mg BD, rivaroxaban 15 mg OD, apixaban 2.5 mg BD) is mandatory in patients with moderate kidney dysfunction (creatinine clearance between 30-50 ml/min) [50]. The only DOAC considered to be reliable is apixaban [47]. VKAs are preferred over DOACs in renal impairment [49].

Limitations

This article focuses on the role of DOACs and their efficacy compared to warfarin in preventing strokes in non-valvular AF. It does not consider using other anticoagulants, such as heparin, and antiplatelet agents, like aspirin and clopidogrel. The use of DOACs in other thrombotic states, such as pulmonary embolism (PE), deep vein thrombosis (DVT), the perioperative period, and cardioversion, has yet to be covered in this study.

## Conclusions

Through this review, we concluded that DOACs exemplify an alluring alternative to VKA therapy for the prevention of stroke or systemic embolism in individuals with atrial fibrillation. Anticoagulation remains at the forefront of the prevention of stroke. The significant implication of this study was the efficacy and safety of DOACs, which did not depend on the adequacy of warfarin management. These drugs exhibited a quicker onset of action, faster absorption, fewer food or drug interactions, no bridging required with other anticoagulants, and did not demand frequent anticoagulant monitoring; hence, they were preferred in patients with AF. However, bleeding complications, especially intracranial hemorrhage, were found to be significantly lower with this group of drugs, except for gastrointestinal (GI) bleeding. Despite the intense curiosity surrounding DOACs, certain conditions, such as the patient's age, bleeding risk, and liver and renal function, should be evaluated before prescribing these agents. One must implement the CHA2DS2-VASc score for assessing an individual's risk of developing stroke in AF, and further attempts to evaluate stroke risk prediction and stratification are needed. Therefore, the main aim of a clinician should be to focus on early stroke risk determination and follow a pragmatic approach while managing patients with AF. Lastly, we firmly believe extensive research analyses are mandated on the role of DOACs for stroke prevention in AF to achieve promising patient outcomes.
